# Completed suicide in bipolar ii patients after their first hospitalization

**DOI:** 10.1192/j.eurpsy.2021.1658

**Published:** 2021-08-13

**Authors:** E. Nieto, A. Palau, C. Russo, P. Alvarez

**Affiliations:** Psychiatry, Althaia XarXa Assistencial of Manresa, MANRESA, Spain

**Keywords:** bipolar II, Suicide, Hospitalization

## Abstract

**Introduction:**

Bipolar disorder, especially Bipolar II subtype, is a mental disorder that has one of the greatest risk of completed suicide (CS).

**Objectives:**

Determine the rate and the risk factors of CS in a cohort of Bipolar II patients followed after their first hospitalization

**Methods:**

We choose all Bipolar II patients (DSM-IV) who were hospitalized for first time in our Psychiatric unit between 1996 and 2016. We reviewed the charts of first hospitalization and recorded multiple baseline variables. In the follow-up we updated the database recording all patients who CS. We compared the different baseline variables between Bipolar II patients who CS and the rest

**Results:**

Of a total of 59 bipolar II patients 6 (10 %) CS in the mean of 13 years of follow up (rate 120 times higher than General Population). The average age at CS was 45.3 years (range between 33 and 57 years old) so there was a 2 years gap on average between the first psychiatric hospitalization and suicide. CS was characterized by a violent act (5 out of 6 cases, 83 %). When we compared BP II patients who CS with the rest, only history of previous violent suicide attempt was detected as a risk factor significantly associated (P<0.04) with CS.

**Conclusions:**

Bipolar II patients CS early after their first hospitalization and at very high rate (120 times than GP) almost always by violent method. History of previous violent suicide attempt is predictor of completed suicide

**Disclosure:**

No significant relationships.

